# Yet Another Similarity between Mitochondrial and Bacterial Ribosomal Small Subunit Biogenesis Obtained by Structural Characterization of RbfA from *S. aureus*

**DOI:** 10.3390/ijms24032118

**Published:** 2023-01-20

**Authors:** Aydar G. Bikmullin, Bulat Fatkhullin, Artem Stetsenko, Azat Gabdulkhakov, Natalia Garaeva, Liliia Nurullina, Evelina Klochkova, Alexander Golubev, Iskander Khusainov, Natalie Trachtmann, Dmitriy Blokhin, Albert Guskov, Shamil Validov, Konstantin Usachev, Marat Yusupov

**Affiliations:** 1 Laboratory of Structural Biology, Institute of Fundamental Medicine and Biology, Kazan Federal University, 420021 Kazan, Russia; 2 Department of Integrated Structural Biology, Institute of Genetics and Molecular and Cellular Biology, University of Strasbourg, 67400 Illkirch, France; 3 Institute of Protein Research, Russian Academy of Sciences, 142290 Pushchino, Russia; 4 Groningen Biomolecular Sciences and Biotechnology Institute (GBB), University of Groningen, 9700 AB Groningen, The Netherlands; 5 Open Lab ‘Biomarker’, Kazan Federal University, 420008 Kazan, Russia; 6 Institute of Microbiology, University of Stuttgart, D-70569 Stuttgart, Germany; 7 NMR Laboratory, Medical Physics Department, Institute of Physics, Kazan Federal University, 420008 Kazan, Russia; 8 Federal Research Center “Kazan Scientific Center of Russian Academy of Sciences”, 420111 Kazan, Russia

**Keywords:** RbfA, 30S biogenesis, ribosome, translation, bacterial ribosomal proteins, *Staphylococcus aureus*

## Abstract

Ribosome biogenesis is a complex and highly accurate conservative process of ribosomal subunit maturation followed by association. Subunit maturation comprises sequential stages of ribosomal RNA and proteins’ folding, modification and binding, with the involvement of numerous RNAses, helicases, GTPases, chaperones, RNA, protein-modifying enzymes, and assembly factors. One such assembly factor involved in bacterial 30S subunit maturation is ribosomal binding factor A (RbfA). In this study, we present the crystal (determined at 2.2 Å resolution) and NMR structures of RbfA as well as the 2.9 Å resolution cryo-EM reconstruction of the 30S–RbfA complex from *Staphylococcus aureus* (*S. aureus*). Additionally, we show that the manner of RbfA action on the small ribosomal subunit during its maturation is shared between bacteria and mitochondria. The obtained results clarify the function of RbfA in the 30S maturation process and its role in ribosome functioning in general. Furthermore, given that *S. aureus* is a serious human pathogen, this study provides an additional prospect to develop antimicrobials targeting bacterial pathogens.

## 1. Introduction

The ribosome is a key element in the central dogma of molecular biology and is thus the focus of pharmacologic research. With the escalating issue of resistance to antibiotics targeting the active translation process, immature ribosomes offer a promising source for the advancement of antibiotics [1,2,3,4,5]. Ribosome biogenesis (maturation) is a complex and highly conservative process in all living organisms. The small and large ribosomal subunits are assembled in parallel almost independently from each other, followed by association into active monosomes [6,7]. Subunit assembly consists of a number of strictly coordinated events including transcription of ribosomal RNA (rRNA), its post-transcriptional modification and processing, and translation and modification of ribosomal proteins, followed by their cooperative association [8,9,10].

The high fidelity of subunit assembly is sustained by a number of auxiliary protein factors, such as RNAses, helicases, GTPases, chaperones, RNA- and protein-modifying enzymes, and assembly factors. Their exact functions, precise localization, and timing are not entirely understood. Nevertheless, it is generally accepted that assembly factors help to avoid kinetic traps during rRNA folding, facilitate the binding of ribosomal proteins, and prevent their premature and non-native binding [11,12,13,14,15,16].

One such assembly factor involved in small ribosomal subunit maturation is the ribosomal binding factor A (RbfA). Its homologs are found in most eubacteria and archaebacteria, and plant and algae chloroplasts, as well as in the mitochondria of eukaryotes. In general, RbfA is a small, compact, single-domain protein with a molecular weight of 13–15 kDa [17,18,19,20]. All of the RbfA homologs have an affinity for the mature small subunit and its assembly intermediates but not for the monosome or polysome.

Initially, RbfA was identified as a cold-shock protein involved in the adaptation of cells to low temperatures [21,22]. When the ambient temperature drops to 10–15 °C, cell growth slows down with a decrease in the overall level of protein biosynthesis and an increase in the number of inactive ribosomes. The adaptation of cells to low temperatures is supervised first by the so-called major cold shock proteins, and then by supporting cold shock proteins such as RbfA and other ribosome maturation factors. A small amount of RbfA is found in cells under normal conditions, but its quantity increases sharply during cold shock. Cells lacking the *rbfA* gene accumulate 17S rRNA intermediates, show disruption of 70S assembly with an increased number of individual 30S and 50S subunits, and lose viability at 15 °C. RbfA is a suppressor for the cold sensitivity of a C23U mutation in helix 1 (h1) on 16S rRNA *5*′-end [23,24].

RbfA proteins have a *KH*-domain-(type II)-like fold which is characteristic of RNA- and ssDNA-binding proteins [25,26,27]. The helices α2 and α3 of the *KH*-domain form a helix–kink–helix (hkh) structure with a sequence motif (GXXG but AXG in RbfA) including a highly conserved Ala residue at the turn forming an interhelical kink [20]. Mitochondrial RbfA has long *C*- and *N*-terminal extensions with additional functions for small subunit maturation [28].

The RbfA facilitates the correct folding of the functional core of rRNA and is required for 30S maturation and translation initiation. At first, it was assumed that RbfA was needed for the correct processing of the 16S rRNA *5*′-end during 30S ribosome subunit maturation [24]. However recent data obtained by cryo-electron microscopy (cryo-EM) and isotope labeling have revealed that RbfA binds to the small subunit after the transition of 16S rRNA *3*′-end from the mRNA entry position to the exit position during the last stages of 30S maturation. This transition is accompanied by reorganization of helix 28 (h28). The RbfA binding site comprises part of the central decoding region (CDR), namely, the h28 (neck), the linker part between the h28/h44, h44/h45 helices, and the *3*′-end of 16S rRNA. The *KH*-domain of RbfA (hkh), as well as the linker part between the α1 and β1 elements, binds to the *3′*-end of rRNA. In addition to interaction with the *3′*-end of rRNA, the loops β1/β2 and α3/β3 of RbfA act as a wedge preventing unwanted tertiary interactions in the region between h44/h45, h28, and the *3′*-end. All of these lead to the stabilization of the subunit neck and its transition from the immature position to the correct mature state [29]. It has been suggested that RbfA can promote formation of the 30S central pseudoknot (h1 and h2) during earlier stages of maturation by delaying the folding of the h44/h45 linker and thereby allosterically stabilize pseudoknot helices via interaction with adjacent h28 [30]. This is consistent with previous data about the RbfA effect on *5*′-end processing and C23U mutation [23,24].

RbfA bound to 30S prevents entry of immature subunits in the translation cycle and acts as a gatekeeper. RbfA is released from the immature 30S subunit by the GTPase RsgA (YjeQ) and from the mature 30S by the initiation factor 3 (IF3), which promotes translation initiation [31]. It has been recently discovered that a large subunit pseudouridine synthase RluD also contributes to the release of RbfA from the 30S subunit [7]. Numerous structures of RbfA homologs from distinct organisms show high conservation in their functionally important parts which are localized similarly and adopt similar conformation [17,20,24,28,29].

Here, we present the RbfA crystal (2.2 Å resolution) and solution (NMR) structures from *S. aureus* with the cryo-EM structure of the 30S–RbfA complex solved at 2.9 Å resolution. Using a combination of structural biology methods, we determined the precise interactions of RbfA with the 30S subunit from *S. aureus*. We described the universal mode of action of RbfA in bacteria and mitochondria. These results open an interesting route for development of broad-spectrum antibiotics targeting ribosome maturation in microbial pathogens.

## 2. Results and Discussion

### 2.1. General Features of RbfA Homologs and Their Interaction with the Small Ribosomal Subunit

Alignment of RbfA homologs from common pathogens with available structures (PDB ID: 1KKG, 1JOS, 2KZF, 2DYJ) [20,24] showed that the *KH*-domains of the homologs are similar, especially in β1, the α2/α3 kink region, and the α1/β1 linker, whereas the *C*-terminal parts after β3 vary in length and sequence. Comparison of the 30S–RbfA complex from *E. coli* and *S. aureus* revealed identical contacts between RbfA and 16S rRNA of the small ribosomal subunit (Supplementary Materials Figures S1 and S2).

### 2.2. The Crystal Structure of S. aureus RbfA

RbfA was produced heterologously in *E. coli* BL21(DE3) pLysS and purified by metal affinity chromatography (Ni-NTA) and gel filtration. Crystallization was performed with the hanging-drop vapor-diffusion technique. The structure was solved at 2.2 Å resolution by molecular replacement using *H. influenzae* RbfA (PDB ID: 1JOS, 1.7 Å) as a search model. The data collection and refinement statistics are shown in Supplementary Materials Table S1.

**Figure 1:**
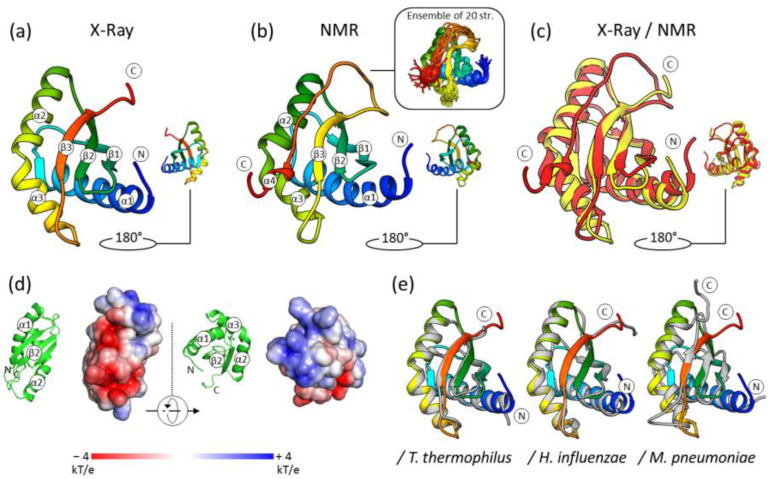
The structure of *S. aureus* RbfA: (**a**) Crystal structure; (**b**) solution NMR structure; (**c**) comparison of crystal (yellow) and NMR (red) structures (Cα RMSD = 1.76 Å); (**d**) electrostatic surface of *S. aureus* RbfA (at pH 7.5); (**e**) comparisons of RbfA structure from *S. aureus* with its homologs from *T. thermophilus* (Cα RMSD = 0.82 Å), *H. influenzae* (Cα RMSD = 0.82 Å), and *M. pneumoniae* (Cα RMSD = 3.32 Å). The homologs’ structures are colored gray.

The crystal structure of *S. aureus* RbfA consists of three α-helices and three β-strands in an α1-β1-β2-α2-α3-β3 order characteristic of RbfA homologs and other proteins with *KH*-domain (type II) organization (Figure 1). Three α-helices are located opposite three antiparallel β-strands, with the following amino acid composition: α1 (4–25), β1 (34–40), β2 (46–52), α2 (57–69), α3 (71–81), and β3 (89–94). The α2 and α3 helices are located at an angle (~120°) with strongly conserved Ala70 at the kink. This is the helix–kink–helix motif (hkh) of the *KH*-domain (the GXXG motif is presented by AXG in RbfA).

The obtained structure reveals the highly conserved structure of RbfA despite the significant amino acid sequence divergence among RbfA homologs (Supplementary Materials Figure S1). Pairwise structure alignment of *S. aureus* RbfA with known structures of homologs revealed a high structural similarity with Cα-RMSD of 1.24 Å, 0.82 Å, 0.82 Å, 3.32 Å, and 1.22 Å for *E. coli*, *T. thermophilus*, *H. influenzae*, *M. pneumonia,* and *T. maritima*, respectively (Figure 1).

RbfA consists mostly of hydrophobic amino acids, which form the hydrophobic core of the molecule between α-helices and the β-sheet. Analysis of the electrostatic surface potential map (at pH = 7.5, as this pH is favorable for ribosomes) revealed a negatively charged region covering the area between α2 and β3 towards β1 via the top of β3 followed by the *C*-terminus (Figure 1). The positively charged region goes along the outer side of α2 to an interhelical kink. The strong positively charged region is formed in between the α2/β3, β1/β2 linkers, and the *N*-terminus of the α1-helix. These features of RbfA organization ensure formation of an extensive interface for interacting with the 16S rRNA core of the 30S subunit.

### 2.3. The Solution NMR Structure of S. aureus RbfA

The NMR structure of the *S. aureus* RbfA was obtained from NMR data (chemical shifts, NOE effects, and dihedral angles) recorded for the double-labeled ^13^C, ^15^N protein. Sequential assignments for the backbone and side-chain resonances have been reported previously [32]. In this study, the NOE analysis of the NMR spectra was carried out and the interproton distances for individual pairs of atoms were determined. The solution NMR structure of RbfA was calculated based on 1486 distance restraints, 85 dihedral constraints, and 57 hydrogen bond constraints (Supplementary Materials Table S2).

The NOE effects of αH–NH (i,i + 3), αH–βH (i,i + 3), and αH–NH (i,i + 4) showed four regular α-helical structures in the Met4–Lys24, Asp57-Lys69, Lys71-Glu79, and His112-Asp115 regions (Supplementary Materials Figure S3). The NOE cross-peaks showed that *S. aureus* RbfA contains three β-strands (Ile33-Leu40, Gln46-Val53, and Glu89-Tyr94). The topology of secondary structure elements is α1-β1-β2-α2-α3-β3-α4. The ensemble view of the NMR structures is shown in Figure 1.

The Ramachandran plot (Supplementary Materials Figure S4) of 10 conformers of RbfA shows that 90.6% of the residues were in the favored regions and 8.7% were in the allowed regions. Thus, the RbfA’s spatial structure was validated by the Ramachandran plot [33].

The functional *KH*-core (α1-β1-β2-α2-α3-β3) of the molecule obtained by NMR is similar to the crystal structure (Cα RMSD = 1.76 Å). Interestingly, the end of the *C*-terminus is determined as a stable small α-helix α4 (His112-Asp115) which has not been observed before (Figure 1). The RbfA secondary structure assignment by the DSSP program revealed a lot of turns and bends (Supplementary Materials Figure S5) for region 95D-118E in NMR structures [34].

We hypothesize that the *C*-terminus after β3 may be predisposed for the formation of an α-helix and it might have a functional role. This hypothesis could be investigated experimentally by the study of the influence of a shortened RbfA without the *C*-terminal helix on 30S subunit maturation.

### 2.4. Cryo-EM Structure of the 30S–RbfA Complex from S. aureus

A reconstitution of the *S. aureus* 30S–RbfA complex was performed by mixing recombinant RbfA and mature 30S subunits, obtained by dissociation of the purified 70S ribosomes. The obtained samples were vitrified and data collection was performed with a 200 kV Talos Arctica microscope equipped with a K2 direct electron detector. The final reconstruction was performed to an overall resolution of 2.9 Å. The local resolution estimation is shown in Supplementary Materials Figure S6.

**Figure 2:**
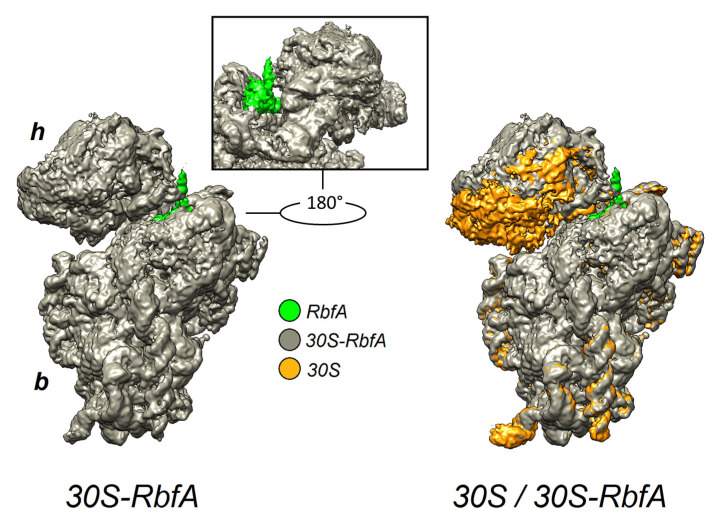
The 2.9 Å cryo-EM density map of the *S. aureus* 30S–RbfA complex (gray) and its comparison with the map of the free *S. aureus* 30S (EMD-3624, orange). The RbfA extra density is colored green. h—head and b—a body of the 30S.

The obtained cryo-EM map allowed unambiguous visualization of the 30S subunit. Comparing the map of the 30S–RbfA complex with individual 30S subunit maps (EMD-23052, EMD-3624) [35,36] revealed an extra density, which was assigned to RbfA. This density is located near the CDR between the head, neck, and central platform of the 30S. The binding site of RbfA on the 30S subunit is in overall agreement with the high-resolution cryo-EM reconstruction obtained for *E. coli* (EMD-12243) [29]. In addition, we noticed that the head of the 30S subunit with RbfA bound is displaced compared to a free 30S (Figure 2), whereas in the rest of the structure we did not observe major conformational changes.

To build the model of the complex we used the RbfA crystal structure obtained in this study and a previously published individual *S. aureus* 30S (PDB ID: 5ND8) [36]. We performed independent fitting of the RbfA, 30S subunit head, and body models into the 30S-RbfA density map in UCSF Chimera [37] followed by real-space refinement in PHENIX [38] and manual adjustments in COOT [39].

It was possible to assign all of the small subunit ribosomal proteins except S21 and S1 in the density. This was also observed recently in a cryo-EM analysis of 30S stabilization in the presence of spermidine [35]. According to the Nomura assembly map [6], S21 is one of the tertiary ribosomal proteins, which binds to the 30S subunit during the late stages of maturation followed by translation initiation and association with 50S. Its binding site overlaps with RbfA [6,40]. S1 is an ‘atypical’ ribosomal protein that binds weakly and transiently associates with the 30S subunit, and it contributes to translation, transcription, and control of RNA stability [41,42].

**Figure 3:**
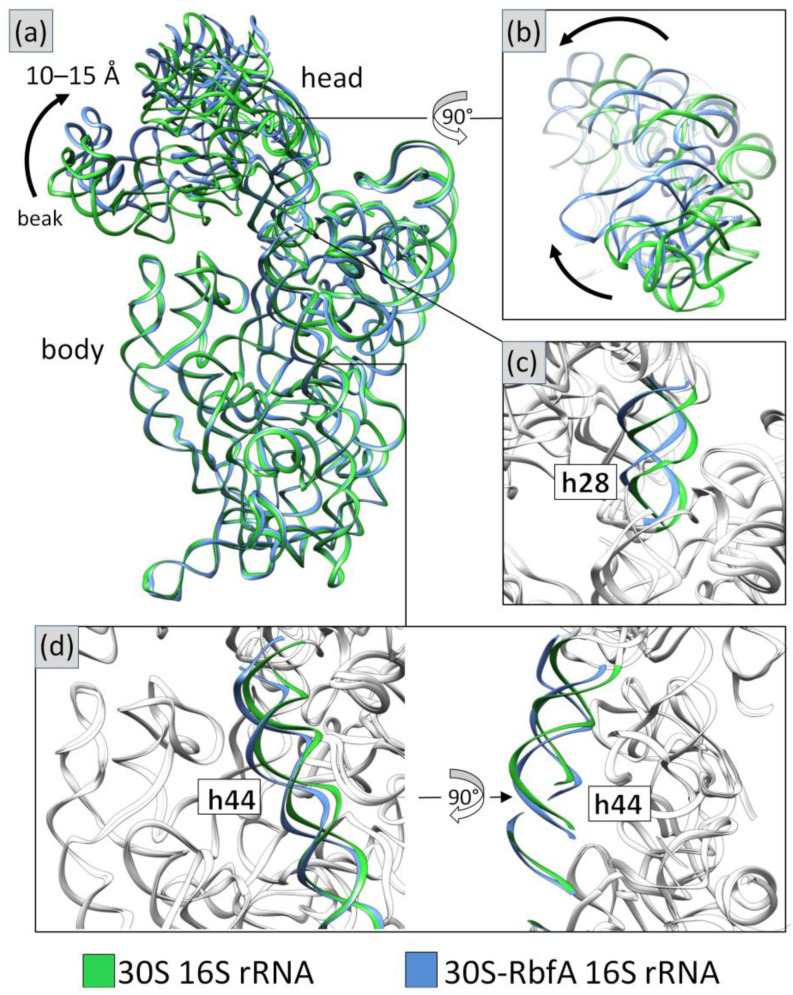
Comparison of 16S rRNA in the mature 30S (PDB ID: 5ND8, green) and 30S–RbfA complex (in this study, blue) from *S. aureus*: (**a**) overall view and (**b**) the head displacement; (**c**,**d**) the conformational changes of helices h28 and h44. The rest of the 16S rRNA is colored white.

The pseudoknot region (h1 and h2) is well resolved, and it shows a stable mature conformation. The 16S *3*′-end is observed inside the mRNA exit channel. h44 is in the front interface position and has a conformation similar to the ***E*** late state of the *E. coli* complex’s assembly (Supplementary Materials Figure S7). The upper part of h44 is shifted to the outer side in comparison with the mature 30S. The h44a conformation described for the *E. coli* complex is not present. h28 has a mature conformation, but the top of the neck is displaced upwards in comparison with the mature subunit. The 30S intermediates from *E. coli* up to the assembly state ***E*** exhibit the same behavior (Supplementary Materials Figure S7). This could indicate the non-stable state of CDR and a link with the head displacement. Therefore, RbfA causes the head to shift relative to the subunit body by displacing the neck. The 30S head moves up and back approximately 10–15 Å from the interface side (Figure 3).

The resolution of the obtained 30S-RbfA map was insufficient to resolve detailed atomic interactions in some regions, especially in the RbfA binding region, although it was possible to fit the main chains of proteins and rRNA, assisted by comparative data analysis obtained by sequence alignment. Given the sequence conservation and the density fit, it seems that the binding mode of RbfA to 30S is very similar in *S. aureus* compared to *E. coli* [29].

**Figure 4:**
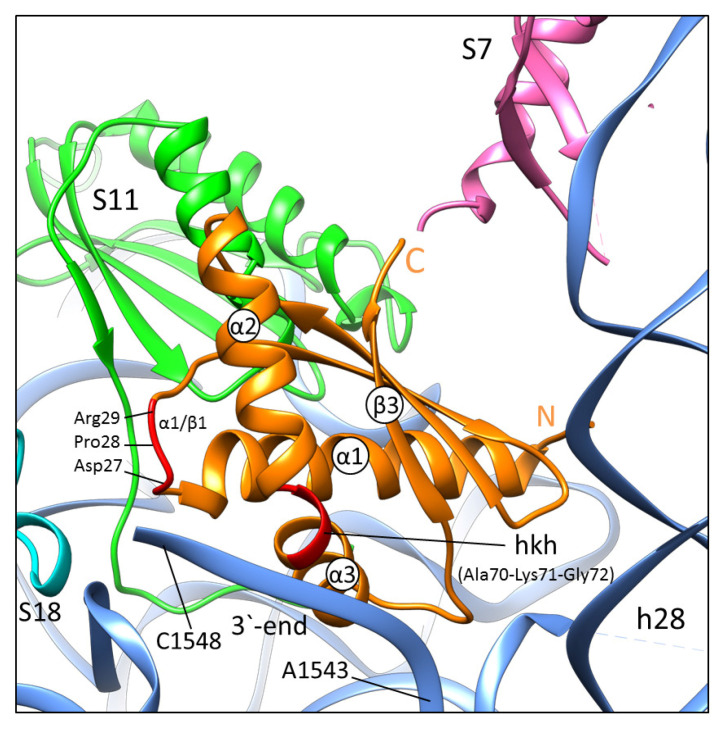
The binding site of RbfA (orange) on the 30S subunit. The RbfA contacting region with 16S rRNA (hkh motif and α1/β1) is colored red. The S7, S11, S18 proteins are colored pink, green, and cyan, respectively. The 16S rRNA is colored blue.

The *3*′-end of 16S rRNA (in the mRNA exit channel) interacts with the hkh motif and the α1/β1 part of RbfA, and the β1/β2 loop is directed toward the cavity between the 30S neck (h28) and the h44-h45 linker. α3/β3 is inserted between two bends of rRNA (G1517 and G1541) and the outer side of α1 approaches the central platform (h23, h24) and S11. The RNA binding side of RbfA (hkh and α1/β1) holds the *3*′-end of 16S rRNA (G1542-A1548) where A1543–C1545 interact with the AXG sequence motif (Ala70-Lys71-Gly72 in *S. aureus* RbfA) of the *KH*-domain. The amino acids Asp27–Arg29 of α1/β1 specifically interact with A1546-C1548 of the *3*′-end, which extends toward the Arg48 of S18 (Figure 4). It is worth noting that in the NMR solution structure of *E. coli* RbfA, the α1/β1 linker contains a 3_10_ helix, which unfolds into a loop upon binding of RbfA to the 30S. We have not observed this additional 3_10_ helix in either of our NMR and crystal structures of RbfA. The conformation of the *KH*-domain in all three structures (NMR, crystal, and complex) is essentially the same.

In our *S. aureus* cryo-EM map, we observed a helix-like density, which was attributed to a part of the *C*-terminal helix of RbfA (Asp95-Arg106). This finding together with observed NMR data suggests that the *C*-terminus could form one more α-helix (α4).

Recent studies of the *M. musculus* complex of mitochondrial RBFA (mtRBFA) (inward conformation) and the small ribosomal subunit have revealed that the bacterial and mammalian homologs share a structural similarity of *KH*-domains [28]. The *M. musculus* mtRBFA has an α-helix following the β3-strand of the *KH*-domain (*KH*-like NTD). Furthermore, it was reported that the association of mtRBFA leads to a vertical shift of uS7m in the head by approximately 12 Å, and the entire head is rotated, compared to the mature state. The data obtained for the *S. aureus* 30S–RbfA complex demonstrate similar head displacement (Figure 3). We hypothesize that the *C*-terminus of *S. aureus* RbfA could adopt a similar conformation and act like the *C*-terminal domain (CTD) of the *M. musculus* mitochondrial homolog.

**Figure 5:**
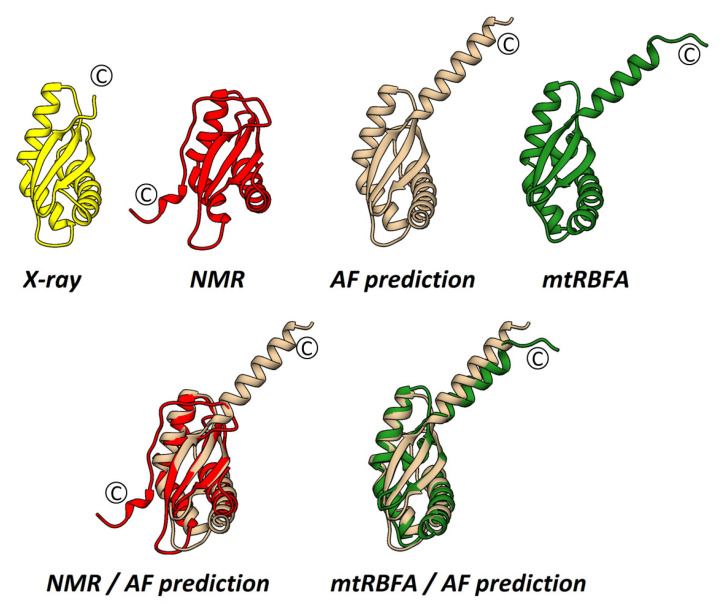
Structure comparisons of *S. aureus* RbfA (crystal—yellow, red—NMR, and grey—AlphaFold prediction) and *M. musculus* mtRBFA (inward conformation without *C*- and *N*-extensions—green). Cα RMSD NMR/AlphaFold = 1.893 Å and Cα RMSD mtRBFA/AlphaFold = 1.123 Å.

We compared the predicted structure of *S. aureus* RbfA from the AlphaFold Protein Structure Database (Uniprot ID: A0A2X3YDX4) [43,44] with the structures obtained experimentally and mtRBFA (PDB ID: 7PNU) (Figure 5). The conformations of the *KH*-domains turned out to be identical for *S. aureus* structures and highly similar to mtRBFA *KH*-like NTD. The whole *C*-terminus following the β3-strand of the predicted structure has an α-helical organization with a per-residue confidence score (pLDDT) of 50–70% or 70–90% in some regions of the terminus.

**Figure 6:**
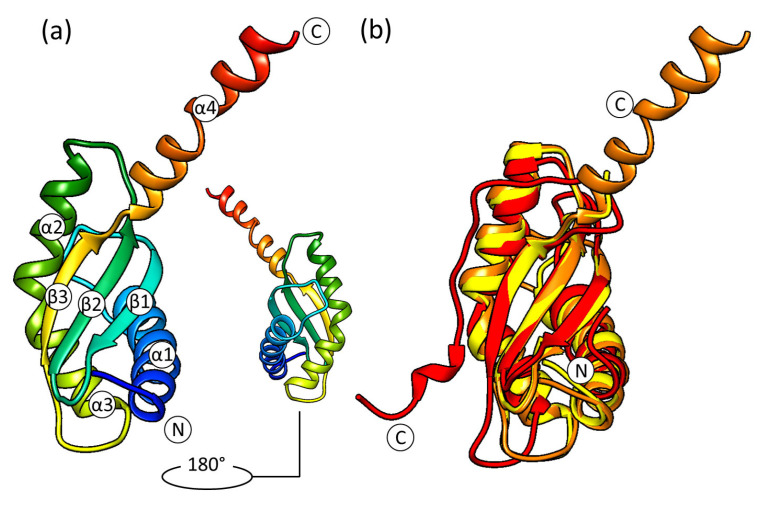
The cryo-EM structure of *S. aureus* RbfA: (**a**) overall view and (**b**) comparison of the cryo-EM structure (orange) with the crystal (yellow, Cα RMSD = 0.69 Å) and NMR (red, Cα RMSD = 1.91 Å) structures.

**Figure 7:**
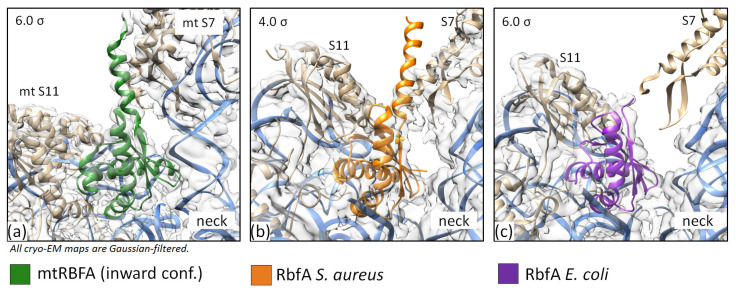
The models and densities of the RbfA *C*-terminus. (**a**) *M. musculus* (PDB ID: 7PNU, EMDB-13552); (**b**) *S. aureus* (this study); and (**c**) *E. coli* (PDB ID: 7BOH, EMD-12243).

Indeed, we found in our cryo-EM map a helix-like extra density following the β3-strand and reaching towards the ribosome head protein S7. This density covers only half of the hypothetical *C*-terminal helix (up to Arg106), but the data obtained by NMR (together with the structure prediction and the mtRBFA structure) indicate the helical organization of the remaining part of the *C*-terminus. We fitted the *C*-terminus of RbfA into this density and built the model with an additional helix (Figure 6). The amino acid residues Ser97-Asp115 of RbfA bound to 30S form a helix-like structure, which interacts with the S7 protein. The map of the *E. coli* 30S–RbfA (EMD-12242) complex also reveals a similar density, albeit it was not described. The comparison of densities for our structure, *E. coli* RbfA, and *M. musculus* mtRBFA is shown in Figure 7.

We suggest that the *C*-terminus helix of RbfA and CTD of mtRBFA share similar functions in preventing mRNA binding to immature small ribosomal subunits. However, the *C*-terminal extension of mtRBFA is suggested to occupy the entire mRNA path [28], while *S. aureus* RbfA is able to block only a part of the mRNA binding region as seen in our cryo-EM structure. Therefore, it is plausible to assume that in bacteria there might be yet another protein, which partly fulfills the function of the *C*-terminal extension of mtRBFA.

**Figure 8:**
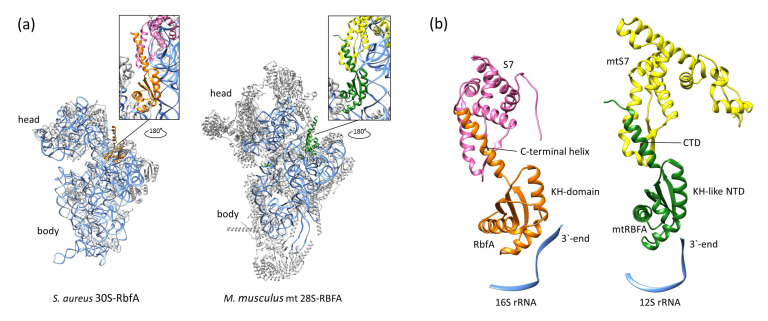
Comparison of bacterial and mitochondrial small subunits bound with RbfA/mtRBFA (PDB ID: 7PNU): (**a**) the localization of RbfA/mtRBFA on small subunits (the rRNAs are colored blue and ribosomal proteins are colored grey) and (**b**) the interactions between RbfA/S7 and mtRBFA/mtS7 proteins.

The fact that we observed different conformations of the *C*-terminus in RbfA in solution, in a crystal, and in a complex is indicative that it has a dynamic nature and can form an α-helix while interacting with S7 of the 30S. The homologs of the S7 head proteins are very conserved, particularly in the region of the last *C*-terminal helix facing RbfA (Supplementary Materials Figure S8). The RbfA and S7 interaction is a good example of the similarity between bacterial and mitochondrial small ribosomal subunits which seem quite different at first glance (Figure 8).

## 3. Materials and Methods

### 3.1. Expression and Purification of RbfA

The *S. aureus rbfA* gene was amplified from genomic DNA using two pairs of specific primers (Supplementary Materials Table S3) and cloned into a pET28a vector with a histidine tag either at the *N*- or the *C*-terminus. The protocols of expression and purification of RbfA for both positions of histidine tags were the same in general. We used RbfA tagged on the *C*-terminus for a crystallization study and RbfA tagged on the *N*-terminus for NMR and cryo-EM structure analysis. Protein expression was carried out in the *E. coli* BL21(DE3) pLysS strain (Novagen) on a selective LB medium or a minimal M9 medium (^13^C-, ^15^N-labeled) [32] containing the appropriate antibiotics. The cells were cultivated in an orbital shaker incubator at 37 °C at 180 rpm. At optical density OD_600_ = 0.6 AU ml^−1^, the expression of the RbfA was induced by the addition of IPTG to a final concentration of 1 mM. The duration of expression was 6 h at 30 °C and 180 rpm (16 h in the case of expression in the M9 medium). Then, the cells were pelleted and stored at –20 °C. After thawing, the cells were disrupted in the basic lysis buffer 1 (20 mM Tris-HCl (pH 7.6), 0.5 M NH_4_Cl, 1 mM DTT) by an endogenous T7 lysozyme (pLysS) and sonication in the presence of a protease inhibitor cocktail (Roche, Basel, Switzerland) and phenylmethylsulfonyl fluoride. The cell lysate was cleared by centrifugation at 25,000× *g* for 30 min and 100,000× *g* for 45 min. Purification of RbfA from the obtained supernatant was performed sequentially by metal chelate affinity chromatography and size exclusion chromatography (SEC). Metal chelate chromatography on Ni-NTA resin (QIAGEN, Hilden, Germany) was carried out in buffer 1, including intermediate salt wash in buffer 2 (20 mM Tris-HCl (pH 7.6), 1 M NH_4_Cl, 1 mM DTT), low imidazole wash in buffer 3 (20 mm Tris-HCl (pH 7.6), 0.5 M NH_4_Cl, 20 mM imidazole, 1 mM DTT), and elution in buffer 4 (20 mM Tris-HCl (pH 7.6), 0.5 M NH_4_Cl, 0.3 M imidazole, 1 mM DTT). Then, the protein was precipitated by (NH_4_)_2_SO_4_ (80%, w/v). SEC was performed using an NGC Discover chromatographic system and an Enrich SEC70 column (BioRad, Hercules, CA, USA) in a buffer containing 0.05 M sodium phosphate (pH 6.8) and 0.25 M NH_4_Cl. The purity of the sample was checked by polyacrylamide gel electrophoresis under denaturing conditions (SDS-PAGE) (Supplementary Materials Figure S9). The obtained samples were concentrated to the required values by Amicon Ultra spin centricons (10 kDa pore size) (Merck, Burlington, MA, USA). The protein concentration was determined spectrophotometrically at 280 nm (the extinction coefficient of RbfA = 4470 M^−1^ cm^−1^).

### 3.2. Crystallization, Data Collection, and Structure Determination

The RbfA crystals were obtained by the hanging-drop vapor-diffusion method. The drops were prepared by mixing 1.3 μL of the protein solution (C_protein_ = 10 mg/mL) with 1.3 μL of reservoir solution (0.1 M MES (pH 6.5), 80 mM manganese (II) chloride, 15%, PEG 20,000). The drops were equilibrated against 0.250 mL reservoir solution at 20 °C. Crystals appeared after 5–7 days and were cryo-protected directly before X-ray data collection by a custom-made cryoprotectant solution (0.17 M ammonium sulfate, 0.085 M sodium acetate trihydrate (pH 4.6), 25.5% PEG 8000, 15% glycerol). Preliminary X-ray data at 100 K were collected using a Cu K-alpha radiation from a PhotonJet-S microfocus sealed tube X-ray generator (Rigaku XtaLAB Synergy-S) equipped with µ-CMF optics (Rigaku Oxford Diffraction) and a HyPix-6000HE detector (Rigaku, Tokyo, Japan). The collection of the high-resolution diffraction data set was performed from a single crystal on BL 14.1 beamline at the Berlin Electron Storage Ring Society for Synchrotron Radiation (BESSY, Berlin, Germany). Diffraction data were collected using a wavelength of 0.992 Å on a DECTRIS PILATUS3 S 6M detector with parameters experimentally optimized based on crystal mosaicity. Data processing was performed by the XDS program package (v. Jun 1, 2017 BUILT=20170923) [45]. The structure was solved by molecular replacement using Phaser from the Phenix package (v. 1.20.1-4487) [38]. RbfA from *H. influenzae* (PDB ID: 1JOS) was used as the starting model. The initially obtained model was refined using phenix.refine (v. 1.20.1-4487) [38], followed by iterative manual building in COOT (v. 0.9.6.EL) [39], and refinement cycles in phenix.refine [38]. The quality of refinement was assessed using the server MolProbity [46]. The data and refinement statistics are presented in Supplementary Materials Table S1. All of the figures were prepared with UCSF Chimera (v. 1.14) [37] and Pymol (v. 1.8) [47].

### 3.3. NMR Characterization

The NMR investigations were performed with 0.9 mM samples of ^13^C, ^15^N-labeled RbfA in PBS buffer (pH 6.8) with 250 mM NH_4_Cl and a mixture of charged amino acids (L-Arg and L-Glu) at 20mM concentration added to the sample to prevent protein aggregation and precipitation [48]. The NMR spectra were carried out on an NMR spectrometer 700 MHz AVANCE III-HD (Bruker, Billerica, MA, USA) with a quadrupole resonance CryoProbe (^1^H/^19^F, ^13^C, ^15^N, ^31^P) at the temperature of 308 K. Assignment of chemical shifts was achieved using standard 3D NMR methods [49]: HNCO, HNCA, HN(CA)CO, HNCACB, CBCA(CO)NH, CC(CO)NH, and HCC(CO)NH for the backbone chain. Assignment of side chain signals was achieved using 3D HCCH-TOCSY и ^1^H–^13^C HSQC-NOESY experiments. The assignments of the ^1^H, ^13^C, and ^15^N backbone and the side-chains’ resonances have been previously reported by our group [32]. Internuclear distances were derived from 2D ^1^H-^1^H NOESY for protein in 100% D_2_O, 3D ^15^N-edited NOESY-HSQC, and 3D ^13^C-edited NOESY-HSQC. Backbone dihedral φ and ψ angles were derived from TALOS+ (v. 3.8) [50]. ^3^J_Ha-NH_ couplings were determined based on 3D HNHA experiments. Experimental restraints (internuclear distances, ^3^J_Ha-NH_ couplings, and dihedral angle restraints) were used for structure calculations by ARIA (v. 2.1) (Ambiguous Restraints for Iterative Assignment) [51]. The mean structure was generated from an ensemble of 10 (out of 1000) water-refined structures with the lowest energy. The ensemble and the structure closest to the mean were then analyzed using PROCHECK (v. 3.4) [52]. Data processing was performed using Bruker Topspin software (v. 3.2). All of the spectra were analyzed using the program CCPNMR (v. 2.5) [53]. Structural figures were generated using UCSF Chimera (v. 1.14) [37].

### 3.4. Ribosome Purification and Dissociation

The protocol described previously [54,55] was used for 70S ribosomes’ purification with minor modifications. In brief, two liters of *S. aureus* RN6390 culture were grown at 37 °C (180 rpm) in a rich nutrient LB medium and harvested at an early logarithmic phase (A_600_ = 1.0 AU mL^−1^). The cells were washed twice with buffer A (20 mM HEPES-KOH (pH 7.5), 100 mM NH_4_Cl, 21 mM Mg(OAc)_2_, and 1 mM EDTA, 1 mM DTT) and pelleted at 4750× *g*. The cell pellet was then frozen at −80 °C. A typical yield was 2–2.5 g of cells per 1 l of LB medium.

For 5 g of cells, the pellet was resuspended in buffer A (30 mL) in the presence of a protease inhibitor cocktail, DNase I (Roche, Basel, Switzerland), and 3.5 mg homemade lysostaphin, followed by lysis at 37 °C for 45 min. The cell lysate was clarified by centrifugation at 30,000× *g* for 90 min. The supernatant was supplemented with 2.8% (w/v) PEG 20,000 (Hampton Research, Aliso Viejo, CA, USA) for the first fractionation step. Then, the concentration of the PEG 20,000 in the recovered supernatant was increased to 4.2% (w/v) for the second fractionation step. The obtained solution was centrifuged at 20,000× *g* for 10 min and the ribosome pellet was resuspended in buffer A (35 mL) and loaded on 25 mL of a sucrose cushion (10 mM HEPES-KOH (pH 7.5), 500 mM KCl, 25 mM Mg(OAc)_2_, 1.1 M sucrose, 0.5 mM EDTA, and 1 mM DTT). Centrifugation was subsequently carried out at 158 420× *g* for 15 h using a Beckman Type 45 Ti rotor (Beckman Coulter Inc., Brea, CA, USA).

The pellet containing the ribosomes was resuspended in buffer E (10 mM HEPES-KOH (pH 7.5), 100 mM KCl, 10 mM Mg(OAc)_2_, 0.5 mM EDTA, and 1 mM DTT) up to a concentration of 7 mg/mL. Then, 500 μL of the sample was loaded onto 7–30% sucrose-density gradients and centrifuged at 17,500 rpm (52 214× *g*) for 15.5 h using a Beckman SW32Ti rotor (Beckman Coulter Inc., Brea, CA, USA). The fractions of the 70S ribosomes were pooled, the concentration of Mg(OAc)_2_ was adjusted to 25 mM, and then PEG 20,000 was added to a final concentration of 4.5% (w/v). The ribosome pellet was obtained by centrifugation at 20,000× *g* for 12 min and then it was gently dissolved in buffer G (10 mM HEPES-KOH (pH 7.5), 50 mM KCl, 10 mM NH_4_Cl, 10 mM Mg(OAc)_2_, and 1 mM DTT) to a final concentration of 25–30 mg/mL. The sample of the 30S was obtained by dissociation of 70S ribosomes in a sucrose gradient (0–30%) in buffer D with a low Mg^2+^ concentration (30 mM NH_4_Cl, 1 mM Mg(OAc)_2_, 10 mM Hepes-K (pH 7.5), and 1 mM DTT) on an Optima XPN-80 ultracentrifuge (Beckman Coulter Inc., Brea, CA, USA) with an SW32Ti rotor at 26,500 rpm (119,730× *g*) for 15 h at 4 °C. Gradient fractions corresponding to the 30S particles were pooled and concentrated using Amicon Ultra spin centricons (30 kDa pore size) (Merck, Burlington, MA, USA) with buffer exchange to buffer G (10 mM NH_4_Cl, 10 mM Mg(OAc)_2_, 10 mM Hepes-K (pH 7.5), 50 mM KCl, 1 mM DTT, and 2.5 mM spermidine). Aliquots of 30 μL with a concentration of ~9 mg/mL were flash-frozen in liquid nitrogen and kept at −80 °C.

### 3.5. Cryo-EM Analysis

A reconstitution of the *S. aureus* 30S–RbfA complex was performed by mixing mature 30S subunits and recombinant RbfA (1:30) by the combined protocol described previously [29,56]. Aliquots of the 2.7 μL 30S-RbfA (~1 mg/mL) complex were applied to freshly glow-discharged holey carbon grids (Quantifoil Au R1.2/1.3 with 2 nm C, 300 mesh), excess liquid was blotted for 4–5 s using a FEI Vitrobot Mark IV (FEI Company, Hillsboro, OA, USA), and the sample was plunge-frozen in liquid ethane at a temperature of approximately 100 K. The TEM grids were transferred into a Talos Arctica 200 keV microscope (Thermo Fisher Scientific, Waltham, MA, USA), equipped with a K2 direct-electron detector (Gatan, Pleasanton, CA, USA). The GIF-quantum energy filter was adjusted to a slit width of 20 eV. A nominal magnification of ×130.000 (yielding a pixel size of 1.022 Å) and a defocus range of −0.5 to −2.0 μm were used for image collection. A total of 876 movie images were collected.

Motion correction, CTF estimation, template-based picking, 2D classification, Ab initio volume generation, and non-uniform 3D refinement were performed using cryoSPARC (v. 4.1) [57]. Maps were sharpened using the Autosharpen Map procedure in Phenix (v. 1.20.1-4487) [38]. The sharpened maps were used for the manual model building using Coot (v. 0.9.6.EL) [39] and refinement of the coordinates was performed in the real space refine module of Phenix [38]. The quality of refinement was assessed using the server MolProbity [46]. Visualization and structure interpretation were carried out in UCSF Chimera (v. 1.14) [37] and PyMol (v. 1.8) [47]. The data and refinement statistics are presented in Supplementary Materials Table S4.

### 3.6. Structure Analysis and Visualization

The multiple sequence alignments were performed by Clustal Omega [58] and UniProt Online tools [59]. Visualization of the images was performed by UCSF Chimera tools (v. 1.14) [37].

## 4. Conclusions

Numerous previous studies have shown the complexity of small ribosomal subunit biogenesis [7,8,9,10,11,12,13,14,15,16,17,18,19,20,21,22,23,24,28,29,30,31,40]. Our data expand on the understanding of RbfA’s function in this process. In summary, we obtained the crystal (2.2 Å) and solution (NMR) structures of *Staphylococcus aureus* RbfA together with the cryo-EM structure of the 30S–RbfA complex solved at 2.9 Å resolution. Structural analysis revealed that *S. aureus* RbfA has a canonical *KH*-domain-like (type II) fold with the following order of secondary structure elements: αββααβ. The *KH*-core of the RbfA is stable, and its conformation in the solution state as well as upon binding to the 30S subunit does not differ from the crystal state. The *S. aureus* RbfA interacts with the 30S subunit in the same manner described for *E. coli*. The *C*-terminus of RbfA bound to the 30S subunit is similar to a mitochondrial *M. musculus* homolog RBFA and forms an additional α-helix interacting with the S7 head protein of 30S. The obtained results clarify RbfA’s function in the process of 30S assembly and highlight the similarity of this process between bacterial and mitochondrial ribosomes.

## Data Availability

The data supporting the findings of this manuscript are available from the corresponding authors upon reasonable request. The coordinates of the isolated *S. aureus* RbfA studied by NMR have been deposited in the PDB with the accession code 6YE5 and its full NMR signal assignments in the BMRB with the accession code 27532. The coordinates of the isolated *S. aureus* RbfA studied by X-ray crystallography have been deposited in the PDB with the accession code 8BXA. For the cryo-EM structure of the *S. aureus* 30S–RbfA complex, coordinates were deposited in the PDB with the accession code 8BYV, while the cryo-EM map was deposited in the electron EMD accession code EMD-16334.

## Supplementary Materials

The following are available online at https://www.mdpi.com/article/10.3390/ijms24032118/s1. References [34,46,49,50,53,58] are cited in the Supplementary Materials.
