# Supplementary material for: Yet Another Similarity between Mitochondrial and Bacterial Ribosomal Small Subunit Biogenesis Obtained by Structural Characterization of RbfA from S. aureus
Source: Int J Mol Sci. 2023 Jan 20;24(3):2118. doi: 10.3390/ijms24032118 (PMC9917171; doi:10.3390/ijms24032118)
Supplement: Supplementary file 1 [file ijms-24-02118-s001.zip › ijms-2159054-supplementary.pdf]

Supplementary Materials

Yet another similarity between mitochondrial and bacterial ribosomal small subunit biogenesis obtained by structural characterization of RbfA from *S. aureus*

Aydar G. Bikmullin, Bulat Fatkhullin, Artem Stetsenko, Azat Gabdulkhakov, Natalia Garaeva, Liliia Nurullina, Evelina Klochkova, Alexander Golubev, Iskander Khusainov, Natalie Trachtmann, Dmitriy Blokhin, Albert Guskov, Shamil Validov, Konstantin Usachev and Marat Yusupov

This file includes:

I. Supplementary Figures S1, S2, S3, S4, S5, S6, S7, S8, S9 with legend;

II. Supplementary Tables S1, S2, S3, S4.

I. SUPPLEMENTARY FIGURES

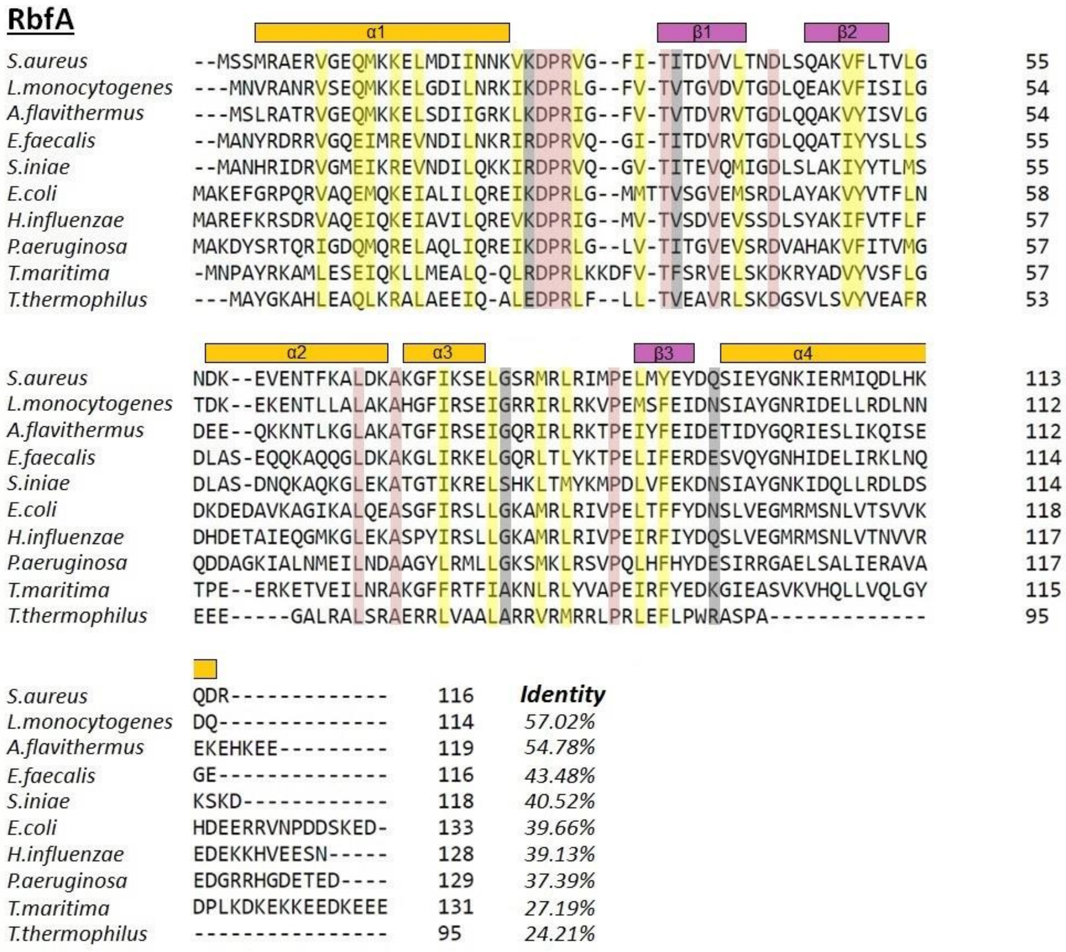

Supplementary Figure S1. Multiple sequence alignment and identity percentages of RbfA from 10 different bacterial species performed in Clustal Omega [58]. Pink shading indicates positions that have a single, fully conserved residue (identity), yellow − conservation between amino acids with substantial sequence identity, and gray − amino acids with low sequence identity. The secondary structure elements of *S. aureus* RbfA are framed in orange and violet.

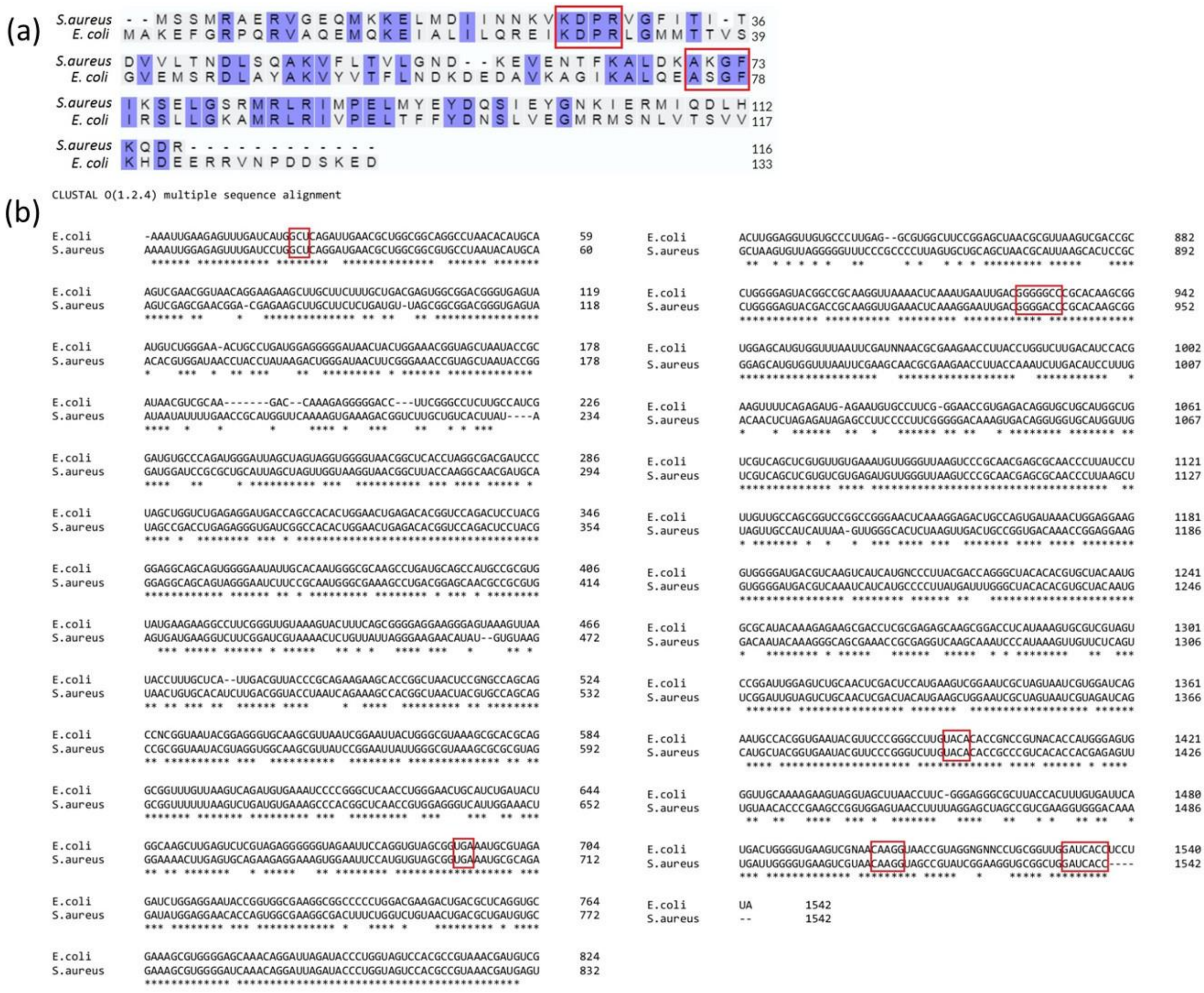

Supplementary Figure S2. Alignment of RbfA amino acid (***a***) and 16S rRNA nucleotide sequences (***b***) from *E. coli* and *S. aureus*. The parts of molecules that form contacts in the complex are framed by red square. An * (asterisk) indicates fully conserved nucleotide positions.

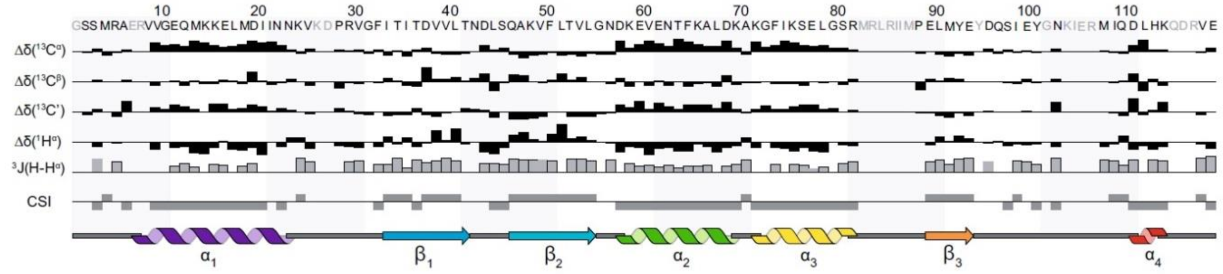

Supplementary Figure S3. The amino acid sequence of *S. aureus* RbfA and TALOS+ secondary structure prediction. Residues shown in gray have not been detected in ^{15}N–^{1}H heteronuclear NMR spectra. The cartoons represent the portions of the RbfA domain predicted by TALOS [49,50] to be α-helical (ribbon) or β-strand (arrow). J coupling value and chemical shift index (CSI) consensus calculated using the CCPNMR [53] drawn as a vertical bar chart.

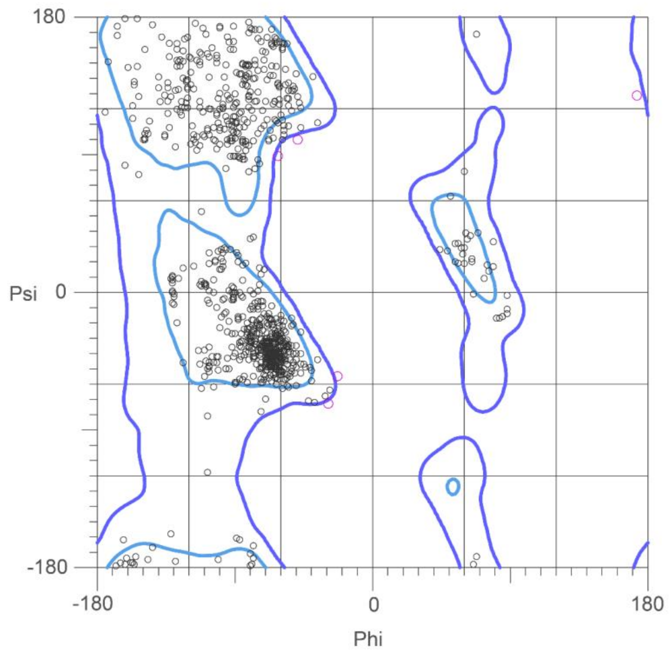

Supplementary Figure S4. The Ramachandran plot of *S. aureus* RbfA conformers.

```
#1
Seq:GSSMRAERVG EQMKKELMDI INNKVKDPRV GFITITDVVL TNDLSQAKVF 50
SS: cccHHHHHHH HHHHHHHHHH HHHHSccSSS ccEEEEEEEE cTTScEEEEE
Seq:LTVLGNDKEV ENTFKALDKA KGFIKSELGS RMRLRIMPEL MYEYDQSIEY 100
SS: EEESScHHHH HHHHHHHHHS HHHHHHHHHT TcScSSccEE EEEEccSSSc
Seq:GNKIERMIQD LHKQDRVE  118
SS: cccScccccc cGGGcccc

#2
Seq:GSSMRAERVG EQMKKELMDI INNKVKDPRV GFITITDVVL TNDLSQAKVF 50
SS: cccHHHHHHH HHHHHHHHHH HHHHSccTTS cccEEEEEEE cTTScEEEEE
Seq:LTVLGNDKEV ENTFKALDKA KGFIKSELGS RMRLRIMPEL MYEYDQSIEY 100
SS: EEcSScHHHH HHHHHHHHHT HHHHHHHHHT TcScScccEE EEEEccSSSc
Seq:GNKIERMIQD LHKQDRVE  118
SS: cccScccccc cGGGcccc

#3
Seq:GSSMRAERVG EQMKKELMDI INNKVKDPRV GFITITDVVL TNDLSQAKVF 50
SS: cccHHHHHHH HHHHHHHHHH HHHHSccTTS ccEEEEEEEE cTTScEEEEE
Seq:LTVLGNDKEV ENTFKALDKA KGFIKSELGS RMRLRIMPEL MYEYDQSIEY 100
SS: EEESScHHHH HHHHHHHHHT HHHHHHHHHT SccSScccEE EEEEccSSSc
Seq:GNKIERMIQD LHKQDRVE  118
SS: ccTTccBccc BGGGGccc

#4
Seq:GSSMRAERVG EQMKKELMDI INNKVKDPRV GFITITDVVL TNDLSQAKVF 50
SS: cccHHHHHHH HHHHHHHHHH HHHHSccTTS ccEEEEEEEE cTTScEEEEE
Seq:LTVLGNDKEV ENTFKALDKA KGFIKSELGS RMRLRIMPEL MYEYDQSIEY 100
SS: EEESScHHHH HHHHHHHHHS HHHHHHHHHT TTcSSSccEE EEEEccSSSc
Seq:GNKIERMIQD LHKQDRVE  118
SS: cccScccScc cTTTTccc

#5
Seq:GSSMRAERVG EQMKKELMDI INNKVKDPRV GFITITDVVL TNDLSQAKVF 50
SS: cccHHHHHHH HHHHHHHHHH HHHHSccTTS ScEEEEEEEE cTTScEEEEE
Seq:LTVLGNDKEV ENTFKALDKA KGFIKSELGS RMRLRIMPEL MYEYDQSIEY 100
SS: EEESScHHHH HHHHHHHHHT HHHHHHHHHS cccSScccEE EEEEccSSSc
Seq:GNKIERMIQD LHKQDRVE  118
SS: cccccccccc cTTTTScc

#6
Seq:GSSMRAERVG EQMKKELMDI INNKVKDPRV GFITITDVVL TNDLSQAKVF 50
SS: cccHHHHHHH HHHHHHHHHH HHHHSccTTS ccEEEEEEEE cTTScEEEEE
Seq:LTVLGNDKEV ENTFKALDKA KGFIKSELGS RMRLRIMPEL MYEYDQSIEY 100
SS: EEESScHHHH HHHHHHHHHT HHHHHHHHHH SSccSSccEE EEEEccSSSc
Seq:GNKIERMIQD LHKQDRVE  118
SS: ccccccSScc cTTTGGGc

#7
Seq:GSSMRAERVG EQMKKELMDI INNKVKDPRV GFITITDVVL TNDLSQAKVF 50
SS: cccHHHHHHH HHHHHHHHHH HHHHSccSSS ccEEEEEEEE cTTSSEEEEE
Seq:LTVLGNDKEV ENTFKALDKA KGFIKSELGS RMRLRIMPEL MYEYDQSIEY 100
SS: EEESScHHHH HHHHHHHHHS HHHHHHHHHT cccSSSccEE EEEEccSSSc
Seq:GNKIERMIQD LHKQDRVE  118
SS: cccScccScc cTTTcScc

#8
Seq:GSSMRAERVG EQMKKELMDI INNKVKDPRV GFITITDVVL TNDLSQAKVF 50
SS: cccHHHHHHH HHHHHHHHHH HHHHSccTTS ScEEEEEEEE cSSScEEEEE
Seq:LTVLGNDKEV ENTFKALDKA KGFIKSELGS RMRLRIMPEL MYEYDQSIEY 100
SS: EEEcScHHHH HHHHHHHHHT HHHHHHHHHH SSccSSccEE EEEEccTTTT
Seq:GNKIERMIQD LHKQDRVE  118
SS: cccSSccccc cSGGGccc

#9
Seq:GSSMRAERVG EQMKKELMDI INNKVKDPRV GFITITDVVL TNDLSQAKVF 50
SS: cccHHHHHHH HHHHHHHHHH HHHTSccSSS ccEEEEEEEE cTTSSEEEEE
Seq:LTVLGNDKEV ENTFKALDKA KGFIKSELGS RMRLRIMPEL MYEYDQSIEY 100
SS: EEESScHHHH HHHHHHHHHS HHHHHHHHHS SSccSSccEE EEEEccSSSc
Seq:GNKIERMIQD LHKQDRVE  118
SS: ccScccSScc cTTTTTTc

#10
Seq:GSSMRAERVG EQMKKELMDI INNKVKDPRV GFITITDVVL TNDLSQAKVF 50
SS: cccHHHHHHH HHHHHHHHHH HHHHSccTTS SccEEEEEEE cTTSSEEEEE
Seq:LTVLGNDKEV ENTFKALDKA KGFIKSELGS RMRLRIMPEL MYEYDQSIEY 100
SS: EEcSScHHHH HHHHHHHHHS HHHHHHHHHH SScSSSccEE EEEEccSSSS
Seq:GNKIERMIQD LHKQDRVE  118
SS: ccSScccccc cSSSSccc
```

Supplementary Figure S5. The *S. aureus* RbfA secondary structure assignment by the DSSP program [34] for region 95D-118E (yellow) in several NMR structures. H – α-helix; B – β-bridge; E – β -strand; G – 310 helix; I – π-helix; T – turn; S – bend; C – coil.

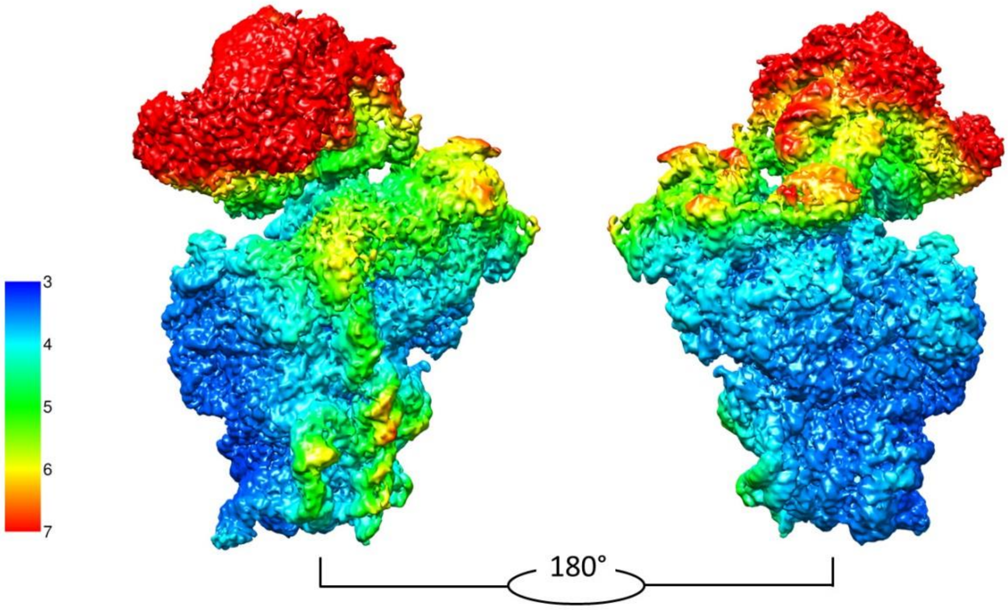

Supplementary Figure S6. Local resolution map of *S. aureus* 30S-RbfA complex.

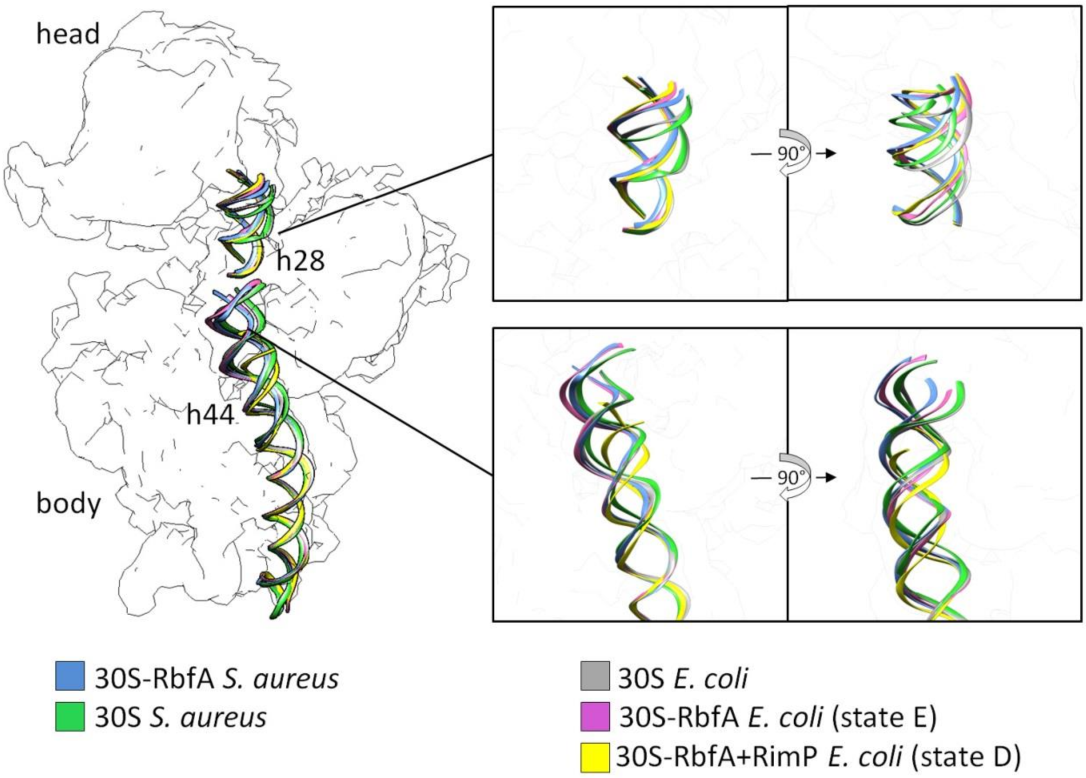

Supplementary Figure S7. Comparison of 16S rRNA h28 and h44 helices of *S. aureus* and *E. coli* in different states (mature or bound with RbfA). Mature S. aureus 30S subunit (PDB ID: 5ND8), mature E. coli 30S subunit (PDB ID: 78 7BOD), E. coli 30S subunit assembly complex state E (PDB ID: 7BOH), E. coli 30S subunit assembly complex state D 79 (PDB ID: 7NAV).

```
CLUSTAL O(1.2.4) multiple sequence alignment


S.aureus            ------------------------------------------------------------      0
E.coli              ------------------------------------------------------------      0
M.musculus mt       MAAPALRAPLRWSGLALGVRCAVWNLPGLTQVRGSRYAPEFREPLIDKEYYRKPVAELTE      60
H.sapiens mt        MAAPAVKVARGWSGLALGVRRAVLQLPGLTQVRWSRYSPEFKDPLIDKEYYRKPVEELTE      60
T. hermophilus      ------------------------------------------------------------      0
H.influenzae        ------------------------------------------------------------      0
M.pneumoniae        ------------------------------------------------------------      0
T.maritima          ------------------------------------------------------------      0


S.aureus            -------MPRKG-SVPKRDVLPDPIHNSKLVTKLINKIMLDGKRGTAQRILYSAFDLVEQ      52
E.coli              -------MPRRR-VIGQRKILPDPKFGSELLAKFVNILMVDGKKSTAESIVYSALETLAQ      52
M.musculus mt       EEKYDQELKKTQFIKAAAATETSSVFADPVISKFTNMMMKGGNKVLARSLMAQTLEAVKR      120
H.sapiens mt        EEKYVRELKKTQLIKAAPAGKTSSVFEDPVISKFTNMMMIGGNKVLARSLMIQTLEAVKR      120
T.thermophilus      -------MARRR-RAEVRQLQPDLVYGDVLVTAFINKIMRDGKKNLAARIFYDACKIIQE      52
H.influenzae        -------MPRRR-SVEARKILPDPKFGSELLAKFINVIMVDGKKSVAESIVYGALETLAQ      52
M.pneumoniae        --------MRKN-RAPKRTVLPDPVFNNTLVTRIINVIMEDGKKGLAQRILYGAFDLIEQ      51
T.maritima          --------MRRR-RAEKRQIPPDPVFGDVLVAKLINRVMWDGKKTIAQKIVYGAFDIIRE      51
                             :            .  . . ::: : * :* .*::  *  :.  : . : .

S.aureus            ---------------RSGRDALEVFEEAINNIMPVLEVKARRVGGSNYQVPVEVRPERRT      97
E.coli              ---------------RSGKSELEAFEVALENVRPTVEVKSRRVGGSTYQVPVEVRPVRRN      97
M.musculus mt       KQFEKYRAASAEEQATIERNPYRIFHEALKNCEPVIGLVPILKGGHFYQVPVPLADRRRR      180
H.sapiens mt        KQFEKYHAASAEEQATIERNPYTIFHQALKNCEPMIGLVPILKGGRFYQVPVPLPDRRRR      180
T.thermophilus      ---------------KTGQEPLKVFKQAVENVKPRMEVRSRRVGGANYQVPMEVSPRRQQ      97
H.influenzae        ---------------RTGKEPLEAFEVALENVRPTVEVKSRRVGGSTYQVPVEVRPVRRN      97
M.pneumoniae        ---------------RTKEKPLTVFERAVGNVMPRLELRVRRIAGSNYQVPTEVPQDRKI      96
T.maritima          ---------------KTKKDPLEVFRQAVENVKPVLEVRPRRVGGATYQVPIEVQEPRRT      96
                                      ..    *. *: *  * : :     .*  ****  :   *:

S.aureus            TLGLRWLVNYARLRGE--KTMEDRLANEILDAANNTGGAVKKREDTHKMAEANKAFAHYR      155
E.coli              ALAMRWIVEAARKRGD--KSMALRLANELSDAAENKGTAVKKREDVHRMAEANKAFAHYR      155
M.musculus mt       FLAMKWMITECRENKPRRTLMPEKLSHELLEAFHNRGPVIKRKHNMHKMAEANRALAHYR      240
H.sapiens mt        FLAMKWMITECRDKKHQRTLMPEKLSHKLLEAFHNQGPVIKRKHDLHKMAEANRALAHYR      240
T.thermophilus      SLALRWLVQAANQRPE--RRAAVRIAHELMDAAEGKGGAVKKKEDVERMAEANRAYAHYR      155
H.influenzae        ALGMRWIVEAARKRGD--KSMALRLANELSDASDNKGAAVKKREDVHRMAEANKAFAHYR      155
M.pneumoniae        ALALRWIAMFARKRHE--KTMLEKIANEIIDASNNTGAAIKKKDDTHKMAEANKAFAHMR      154
T.maritima          SLALRWIVEAARAKKG--RPMKEKLAEEIIAAYNNTGTAIKKKEDTHRMAEANRAFAHYR      154
                     *.::*:   .. .         :::.::  * .. * .:*::.: .:*****:* ** *

S.aureus            W-----------------------      156
E.coli              WLSLRSFSHQAGASSKQPALGYLN      179
M.musculus mt       WW----------------------      242
H.sapiens mt        WW----------------------      242
T.thermophilus      W-----------------------      156
H.influenzae        W-----------------------      156
M.pneumoniae        W-----------------------      155
T.maritima          W-----------------------      155
                    *
```

Supplementary Figure S8. Comparison of homologs of S7 ribosomal protein from different organisms was performed in Clustal Omega [58]. Pink shading indicates positions that have a single, fully conserved residue (identity), yellow − conservation between amino acids with substantial sequence identity. The helix of S7 interacting with the *C*-terminal helix of RbfA is framed in red. An * (asterisk) indicates positions which have a single, fully conserved residue. A : (colon) indicates conservation between groups of strongly similar properties - scoring > 0.5 in the Gonnet PAM 250 matrix. A . (period) indicates conservation between groups of weakly similar properties - scoring =< 0.5 in the Gonnet PAM 250 matrix [58].

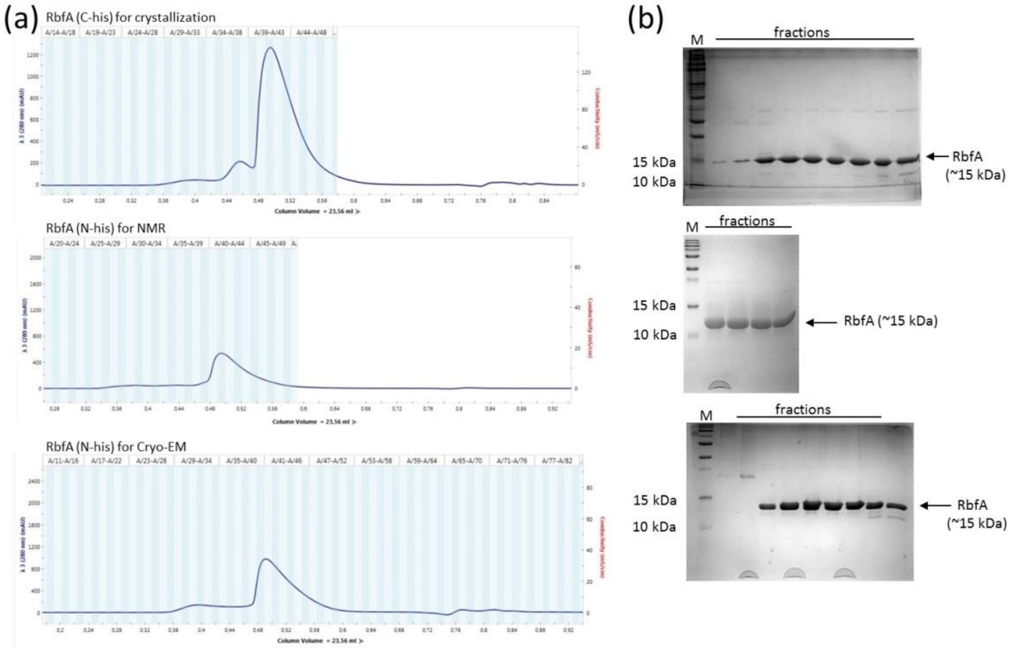

Supplementary Figure S9. Purification of the *S. aureus* RbfA: (***a***) Size exclusion chromatography (SEC) profiles of the *S. aureus* RbfA; (***b***) Analysis of the selected fractions after SEC by 15% SDS PAGE. The first lane is the protein ladder (M).

II. SUPPLEMENTARY TABLES

Supplementary Table S1. X-ray data collection and refinement statistics.

	RbfA
Resolution range (Å)	32.66 - 2.22 (2.3 - 2.22)
Space group	P 4_1
Unit cell	
a, b, c (Å)	65.31, 65.31, 27.28
α, β, γ (°)	90.0, 90.0, 90.0
Multiplicity	7.2 (6.9)
Completeness (%)	99.88 (100.00)
Mean I/sigma(I)	18.60 (2.44)
Wilson B-factor	49.47
R-merge	0.06 (0.78)
R-meas	0.06 (0.85)
R-pim	0.02 (0.32)
$CC_{1/2}$	0.99 (0.89)
Refinment	
R-work	0.20
R-free	0.24
No. of atoms	852
protein	838
water	14
R.m.s. deviations	
Bond lengths (Å)	0.01
Bond angles (°)	1.01
Ramachandran favored (%)	98.97
Ramachandran allowed (%)	1.03
Clashscore	9.94
Average B-factor	56.79
protein	56.81
water	55.68

Statistics for the highest-resolution shell are shown in parentheses.

Supplementary Table S2. Statistical information for RbfA structural ensemble.

Distance Constraints	
total	1486
intra-residual	372
inter-residual	1114
sequential (i - j = 1)	495
medium (i - j = 2,3,4)	388
long-range (i - j > 4)	231
Dihedral Angle Constraints	
Phi restraints	85
Psi restraints	85
Structural Statistics	
number of NOE violations	10
number of dihedral angle restraint violations	0
RMSD for bond deviations (Å)	0.01
RMSD for angle deviations (deg)	0.99
RMSD of all backbone atoms (Å)	
Met4-Tyr94	1.02 ± 0.26
Met4-Lys24	0.44 ± 0.12
Asp57-Lys69	0.22 ± 0.11
Lys71-Glu79	0.28 ± 0.09
His112-Asp115	0.75 ± 0.37
Ile33-Leu40	0.34 ± 0.12
Gln46-Val53	0.25 ± 0.09
Glu89-Tyr94	0.30 ± 0.10
Ramachandran Plot	
residues in the most favored region (%)	90.6
residues in the additionally allowed region (%)	8.7
residues in the disallowed region (%)	0.7

Supplementary Table S3. Oligonucleotide primers for *S. aureus* RbfA cloning.

Primer name	Primer sequence
rbfA_Nhis_NdEI_fwd	5'-TTTTTTCATATGAGCAGTATGAGAGCAGAGCGTGT-3'
rbfA_Nhis_HindIII_rev	5'-TTTTTTAAGCTTATCTATCTTGTTTGTGTAAATCTTGAATCATTCGTTCAA-3'
rbfA_Chis_NcoI_fwd	5'-TTTTTTCCATGAGCAGTATGAGAGCAGAGCG-3'
rbfA_Chis_XhoI_rev	5'-TTTTTTCTCGAGTCTATCTTGTTTGTGTAAATCTTGAATCATTCGTTCAA-3'

Supplementary Table S4. Data collection, refinement, and validation statistics for *S. aureus* 30S-RbfA complex cryo- EM structure.

Data collection	
Microscope	FEI TALOS ARCTICA
Camera	Gatan K2 Quantum (4k x 4k)
Voltage (kV)	200
Defocus range (μm)	(-1.5) – (-3.0)
Total dose (ē/Å2)	53.3
Dose per frame(ē/Å^2)	0.89
Number of frames	60
Image processing	
Number of images	876
Number of particles (used for 3D reconstruction)	81421
Resolution (Å; at FSC[a] = 0.143)	2.89
CC[a] (mask)	0.70
CC (box)	0.71
Validation[b]	
Clashscore[c]	15.08
Proteins	
MolProbity score	1.97
Poor rotamers (%)	0.93
Favored rotamers (%)	70.51
Ramachandran allowed(%)	4.18
Ramachandran favored (%)	95.82
RMS deviations	
Bonds (Å)	0.005
Angles (°)	0.712
Bad bonds (%)	0.00
Bad angles (%)	0.00
Cis-Prolines (%)	0.00
CaBLAM outliers (%)	3.8
CA Geometry outliers (%)	0.83
RNA	
Probably wrong sugar puckers (%)	0.26
Bad backbone conformations (%)	17.35
Bad bonds (%)	0.00
Bad angles (%)	0.00

[a] FSC, Fourier shell correlation; [b] Compiled using MolProbity [46]; C[C], correlation coefficient; RMS, root-mean square.
